# Doxycycline down-regulates DNA-PK and radiosensitizes tumor initiating cells: Implications for more effective radiation therapy

**DOI:** 10.18632/oncotarget.4159

**Published:** 2015-06-13

**Authors:** Rebecca Lamb, Marco Fiorillo, Amy Chadwick, Bela Ozsvari, Kimberly J. Reeves, Duncan L. Smith, Robert B. Clarke, Sacha J. Howell, Anna Rita Cappello, Ubaldo E. Martinez-Outschoorn, Maria Peiris-Pagès, Federica Sotgia, Michael P. Lisanti

**Affiliations:** ^1^ The Breakthrough Breast Cancer Research Unit, Institute of Cancer Sciences, University of Manchester, UK; ^2^ The Manchester Centre for Cellular Metabolism (MCCM), Institute of Cancer Sciences, University of Manchester, UK; ^3^ The Department of Pharmacy, Health and Nutritional Sciences, The University of Calabria, Italy; ^4^ The Cancer Research UK Manchester Institute, University of Manchester, UK; ^5^ The Sidney Kimmel Cancer Center, Philadelphia, PA, USA

**Keywords:** doxycycline, mitochondrial biogenesis, radiation resistance, proteomic analysis, DNA-PK

## Abstract

DNA-PK is an enzyme that is required for proper DNA-repair and is thought to confer radio-resistance in cancer cells. As a consequence, it is a high-profile validated target for new pharmaceutical development. However, no FDA-approved DNA-PK inhibitors have emerged, despite many years of drug discovery and lead optimization. This is largely because existing DNA-PK inhibitors suffer from poor pharmacokinetics. They are not well absorbed and/or are unstable, with a short plasma half-life. Here, we identified the first FDA-approved DNA-PK inhibitor by “chemical proteomics”. In an effort to understand how doxycycline targets cancer stem-like cells (CSCs), we serendipitously discovered that doxycycline reduces DNA-PK protein expression by nearly 15-fold (> 90%). In accordance with these observations, we show that doxycycline functionally radio-sensitizes breast CSCs, by up to 4.5-fold. Moreover, we demonstrate that DNA-PK is highly over-expressed in both MCF7- and T47D-derived mammospheres. Interestingly, genetic or pharmacological inhibition of DNA-PK in MCF7 cells is sufficient to functionally block mammosphere formation. Thus, it appears that active DNA-repair is required for the clonal expansion of CSCs. Mechanistically, doxycycline treatment dramatically reduced the oxidative mitochondrial capacity and the glycolytic activity of cancer cells, consistent with previous studies linking DNA-PK expression to the proper maintenance of mitochondrial DNA integrity and copy number. Using a luciferase-based assay, we observed that doxycycline treatment quantitatively reduces the anti-oxidant response (NRF1/2) and effectively blocks signaling along multiple independent pathways normally associated with stem cells, including STAT1/3, Sonic Hedgehog (Shh), Notch, WNT and TGF-beta signaling. In conclusion, we propose that the efficacy of doxycycline as a DNA-PK inhibitor should be tested in Phase-II clinical trials, in combination with radio-therapy. Doxycycline has excellent pharmacokinetics, with nearly 100% oral absorption and a long serum half-life (18–22 hours), at a standard dose of 200-mg per day. In further support of this idea, we show that doxycycline effectively inhibits the mammosphere-forming activity of primary breast cancer samples, derived from metastatic disease sites (pleural effusions or ascites fluid). Our results also have possible implications for the radio-therapy of brain tumors and/or brain metastases, as doxycycline is known to effectively cross the blood-brain barrier. Further studies will be needed to determine if other tetracycline family members also confer radio-sensitivity.

## INTRODUCTION

Recently, we employed a label-free quantitative proteomics approach to discover new therapeutic targets in tumor-initiating cells (TICs) and/or cancer stem-like cells (CSCs) [1]. Briefly, ER-positive breast cancer lines were cultured under suspension conditions, allowing for the formation of mammospheres (a.k.a., tumor-spheres). Then, we compared the proteome of these tumor-spheres directly to attached monolayer cells grown in parallel. Using this approach, we identified several functional groups of proteins that were specifically up-regulated in tumor-spheres, relative to epithelial monolayers. More specifically, mitochondrial proteins were dramatically over-expressed, including key enzymes related to beta-oxidation and ketone metabolism, as well as proteins involved in mitochondrial biogenesis, and specific protein inhibitors of mitophagy [1]. Interestingly, our approach revealed > 60 mitochondrial-related target proteins that were significantly over-expressed in tumor-spheres. These mitochondrial proteins were also transcriptionally over-expressed in human breast cancer samples, highlighting their potential clinical importance. As such, we believe that increased mitochondrial biogenesis could provide a new mechanism for the accumulation of mitochondrial mass in CSCs.

We also utilized a pharmacological approach to functionally validate the importance of mitochondrial fuels, by employing a specific MCT1/2 inhibitor, which prevents the uptake of two mitochondrial fuels, ketone bodies and L-lactate. Notably, inhibition of MCT function significantly inhibited tumor-sphere formation in both ER-positive and ER-negative breast cancer cell lines, with an IC-50 of ~1–2 μM [1]. Oligomycin A, an inhibitor of Complex V, the mitochondrial ATP-synthase, showed very similar inhibitory activity. Taken together, these results provide suggestive evidence that the proliferative expansion of CSCs strictly depends upon oxidative mitochondrial metabolism [1]. These findings could have future clinical implications for eradicating CSCs, to minimize or prevent tumor recurrence, metastasis and drug resistance. Of course, this would depend on the clinical development of new non-toxic mitochondrial inhibitors that are safe for the treatment of breast cancer patients, which could require 10–15 years of intensive pharmaceutical development and investment.

In summary, using this unbiased proteomic approach, we identified a conserved dependence on mitochondrial biogenesis for the clonal expansion and survival of CSCs. Interestingly, it is well-known that certain FDA-approved antibiotics inhibit mitochondrial biogenesis as a mild, clinically manageably, side-effect [2–6]. Thus, we recently proposed that these antibiotics could be re-purposed as a relatively non-toxic approach to target CSCs. This approach would dramatically accelerate the potential use of mitochondrial inhibitors in cancer patients [7].

Using this strategy, we experimentally identified 5 different classes of mitochondrially-targeted antibiotics that successfully targeted CSCs [7]. Importantly, these FDA-approved drugs inhibited the growth of CSCs in 12 different cancer cell lines, across 8 different tumor types (breast, DCIS, ovarian, prostate, lung, pancreatic, melanoma, and glioblastoma (brain)). The use of generic antibiotics for cancer therapy could significantly reduce the costs of patient care, making treatment more accessible.

Doxycycline is one of the most promising drugs that emerged from these studies [7]. Since its FDA-approval in 1967, it has been successfully used as a broad-spectrum antibiotic for nearly 50 years, without any major side-effects. Currently, acne patients use doxycycline safely for chronic therapy, for up to 6 months at a time, at a standard dose of 200-mg per day [8, 9].

Mechanistically, in mammalian cells, doxycycline functions as an inhibitor of mitochondrial biogenesis, by binding to the small subunit of the mitochondrial ribosome [2–4, 10, 11]. Doxycycline targets the mitochondrial ribosome, as mitochondria evolved from bacteria over millions of years of evolution [12, 13]. Therefore, bacterial ribosomes and mitochondrial ribosomes show many conserved properties and protein homologies. Recent clinical trials with doxycycline (intended to target cancer-associated infections, but not cancer cells) have already shown positive therapeutic effects in lymphoma patients, although their ability to eradicate CSCs was not yet appreciated [14–15].

Here, to better understand the mechanism(s) of action of doxycycline on cancer cell metabolism, we used an unbiased proteomics approach to identify which proteins are effectively down-regulated by doxycycline. This led to identification of doxycycline as the first FDA-approved inhibitor of DNA-PK, which has broad clinical implications for the use of doxycycline as a radio-sensitizer, to overcome radio-resistance in CSCs.

Throughout this paper, we have used the term cancer stem-like cells (CSCs) or tumor-initiating cells (TICs). For a very insightful discussion of the emerging terminology surrounding the definition of CSCs or TICs, please see the following review (16).

## RESULTS

### Doxycycline inhibits mammosphere formation, as assessed using primary breast cancer samples derived from metastatic disease sites

Previously, we established that the antibiotic doxycycline effectively inhibits tumor-sphere formation in 12 different cell lines, across eight different cancer types, including breast cancer. More specifically, both ER-positive (MCF7/T47D) and ER-negative (MDA-MB-231) cell lines were all sensitive to doxycycline treatment [7]. Interestingly, all three of these cell lines were originally derived from the pleural effusions of breast cancer patients, with metastatic disease. Thus, we next tested if primary breast cancer samples derived from metastatic disease sites were also sensitive to doxycycline.

**Figure 1:**
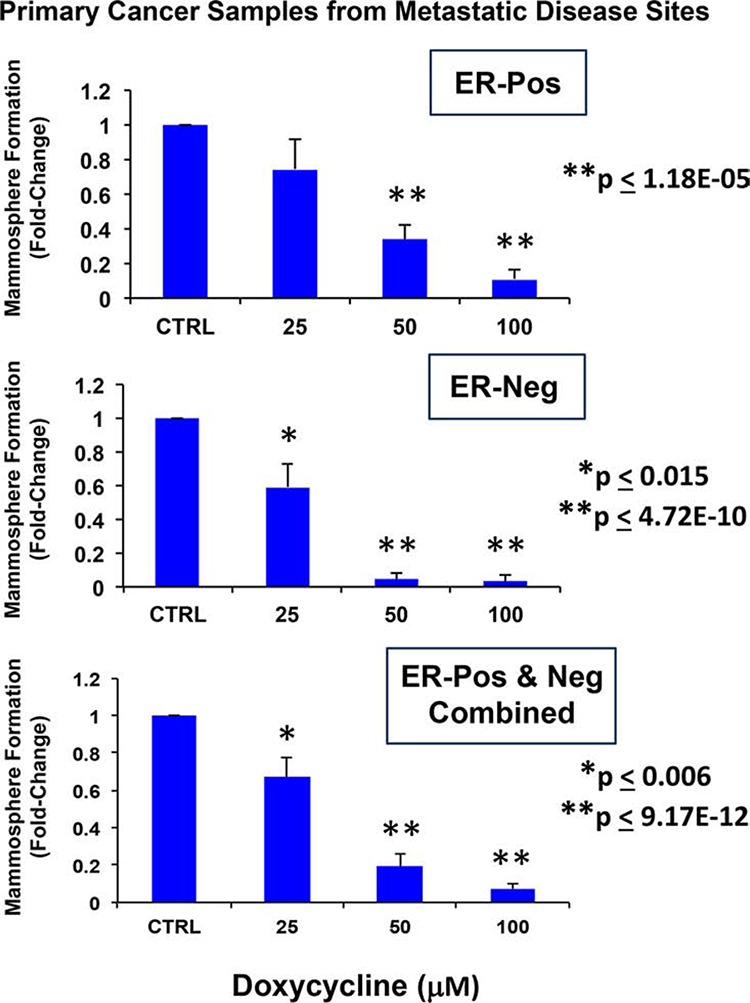
Doxycycline inhibits mammosphere formation, as assessed using primary breast cancer samples derived from metastatic disease sites Upper panel: ER-positive (*N* = 2 patients); Middle panel: ER-negative (*N* = 2 patients); Lower panel: ER-positive and negative samples combined (*N* = 4 patients). Note that doxycycline dose-dependently inhibits mammosphere formation in primary patient's samples derived from metastatic disease sites (either pleural effusions or ascites). Doxycycline appears to work equally well in samples derived from either ER-positive or ER-negative patients. All experiments were performed in triplicate.

Importantly, Figure 1 shows that that doxycycline dose-dependently inhibits mammosphere formation in primary patient samples derived from metastatic disease sites (either pleural effusions or ascites fluid). Moreover, doxycycline appeared to work equally well in samples derived from either ER-positive or ER-negative patients (*N* = 4 patients in total) (See also Supplemental Figure 1). As such, we obtained quantitatively similar results with both well-established cell lines and primary breast cancer samples.

These results are consistent with previous studies showing that doxycycline dramatically inhibits the *in vivo* growth of metastatic lesions (bone and soft tissue) in a mouse model of breast cancer, by up to 60-to-80% [17].

### Doxycycline pre-treatment reduces the mammosphere forming capacity of MCF7 monolayer cells

To better understand how doxycycline inhibits the growth of CSCs, we used an unbiased proteomic approach to identify its potential molecular targets. For this purpose, we established conditions under which doxycycline selectively inhibits the proliferation of CSCs, but not “bulk” cancer cells.

**Figure 2:**
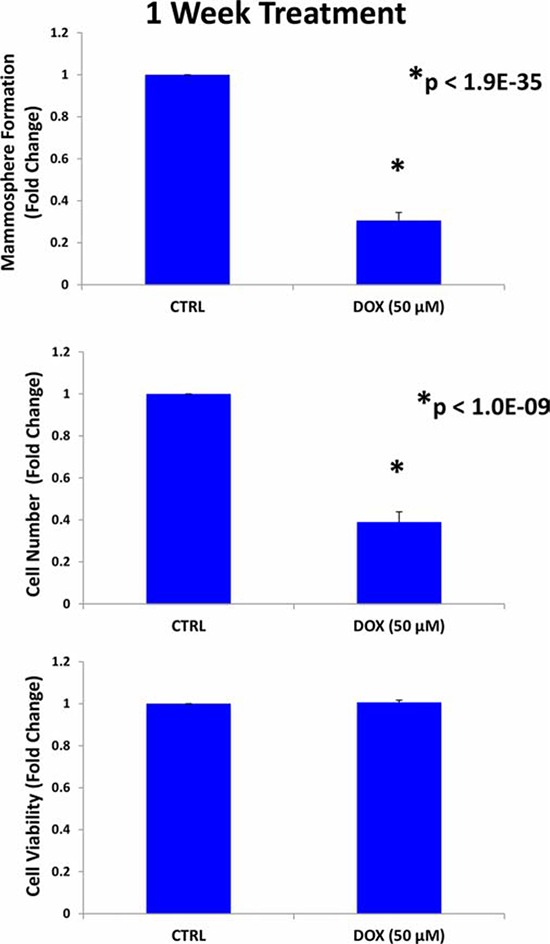
Doxycycline pre-treatment of MCF7 monolayers inhibits mammosphere formation: Effects at 7-days MCF7 cells were pre-treated with doxycycline (50 μM) as monolayers for 7-days and then re-plated for the mammsphere assay, in the absence of doxycycline. Note that pre-treatment with doxycycline, under these conditions, is sufficient to significantly reduce mammosphere forming capacity. However, this 7-day treatment also reduced proliferation in MCF7 cell monolayers to a similar extent, but did not affect the viability of the remaining cells. Each data point in this figure is the average of 9 replicates.

First, MCF7 cells were pre-treated with doxycycline (50 μM) as monolayers for 7-days and then re-plated for the mammosphere assay, in the absence of doxycycline. Figure 2 shows that pre-treatment with doxycycline, under these conditions, is sufficient to significantly reduce mammosphere forming capacity. However, this 7-day treatment also significantly reduced proliferation in MCF7 cell monolayers to a similar extent, but did not affect the viability of the remaining cells.

**Figure 3:**
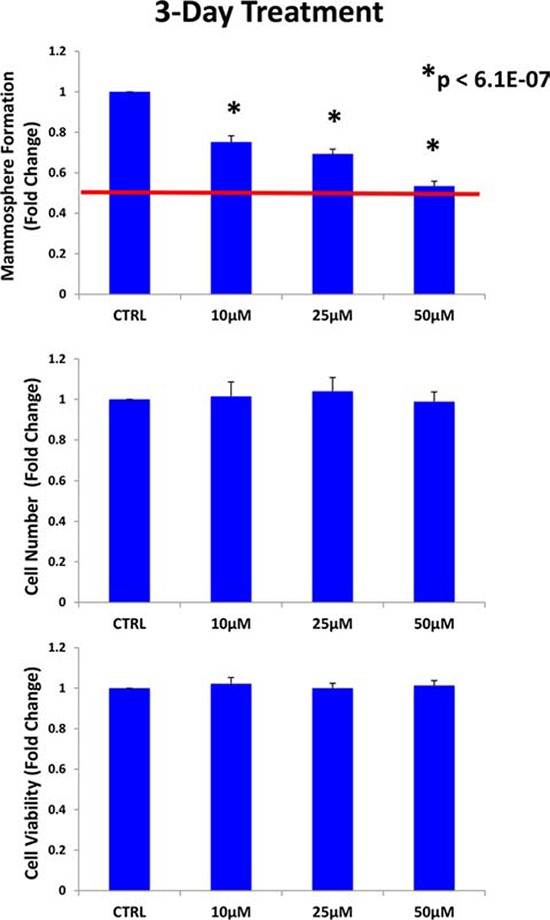
Doxycycline pre-treatment of MCF7 monolayers inhibits mammosphere formation: Effects at 3-days MCF7 cells were pre-treated with doxycycline (50 μM) as monolayers for 3-days and then re-plated for the mammosphere assay, in the absence of doxycycline. Under these conditions, doxycycline (50 μM) reduced the mammosphere forming capacity of MCF7 cells by ~ 50%, without affecting the proliferation of the bulk monolayer cells As such, doxycycline can be used to selectively reduce “stemness” in MCF7 monolayers. Each data point in this figure is the average of 9 replicates.

Therefore, we next shortened the pre-treatment period to 3-days. Importantly, under these new conditions, doxycycline (50 μM) reduced the mammosphere forming capacity of MCF7 cells by ~ 50%, without affecting the proliferation of the bulk monolayer cells (Figure 3). Thus, doxycycline can be used to selectively reduce “stemness” in MCF7 monolayers.

### Identification of the molecular targets of doxycycline, using unbiased label-free proteomics analysis: DNA-PK emerges as a new target

Next, to begin to understand the molecular basis of this selectively, we used these culture conditions to perform unbiased proteomics analysis on MCF7 monolayers (treated with doxycycline for 3-days). The results of this analysis are summarized in Table 1. Only proteins reduced by ≥ 1.5-fold (*p* < 0.05) were considered in the analysis. Importantly, Table 1 clearly highlights that doxycycline pre-treatment of MCF7 cell monolayers significantly reduced the expression of many key protein targets functionally associated with mitochondrial metabolism, glycolysis, the EMT, protein synthesis and the DNA damage response, as well as inflammation and protein degradation, in human breast cancer cells.

**Table 1:** MCF7 cell proteins down-regulated in response to doxycycline treatment of monolayer cultures (3-days at 50 μM)

Symbol	Gene Description	Down-regulation (fold-change)	ANOVA
**Mitochondrial-related Proteins**			
PRKDC	DNA-dependent protein kinase catalytic subunit (maintains mt-DNA integrity & copy number)	14.71	0.02
GPD2	Glycerol-3-phosphate dehydrogenase, mitochondrial	5.29	1.55E-06
MDH2	Malate dehydrogenase 2, mitochondrial	3.08	4.01E-06
ECI1	Enoyl-CoA delta isomerase 1, mitochondrial	2.95	0.018
VDAC1	Voltage-dependent anion-selective channel protein 1	2.71	9.65E-05
HSPD1	60 kDa heat shock protein, mitochondrial	2.24	0.004
DECR1	2,4-dienoyl CoA reductase 1, mitochondrial	1.94	0.02
UQCRC2	Cytochrome b-c1 complex subunit 2, mitochondrial	1.91	0.002
SDHA	Succinate dehydrogenase complex subunit A	1.88	0.0001
ALDH18A1	Delta-1-pyrroline-5-carboxylate synthase, mitochondrial	1.88	0.006
COX6A	Cytochrome c oxidase subunit 6A, mitochondrial	1.84	0.008
FASN	Fatty acid synthase	1.83	0.02
LRPPRC	Leucine-rich PPR-motif containing (inhibitor of mitophagy)	1.72	0.002
ORP150	150 kDa oxygen-regulated protein	1.70	0.02
NDUFA4	NADH dehydrogenase (Ubiquinone) 1 alpha-subcomplex, 4	1.68	0.0007
COX5B	Cytochrome c oxidase subunit 5B, mitochondrial	1.66	0.0008
ATP5F1	ATP synthase, H+ transporting, mitochondrial F0 complex, subunit b	1.57	0.02
**Glycolytic Enzymes**			
GPI	Glucose-6-phosphate isomerase	3.82	8.06E-05
LDHA	L-lactate dehydrogenase A	2.26	0.02
TPI1	Triosephosphate isomerase 1	2.25	0.004
ENO1	Enolase 1	1.71	0.005
ALDOA	Fructose-bisphosphate aldolase A	1.57	0.007
PGK1	Phosphoglycerate kinase 1	1.57	0.037
LDHB	L-lactate dehydrogenase B	1.53	0.03
PKM1/2	Pyruvate kinase	1.45	0.03
**EMT-markers/Muscle-related proteins (cytoskeletal)/Extracellular Matrix/Angiogenesis**			
SMOC2	SPARC-related modular calcium-binding protein 2	7.01	5.17E-06
MYO18B	Unconventional myosin-XVIIIb	6.48	0.0002
TUBB1	Tubulin beta-1 chain	3.72	3.09E-05
ADAM22	A disintegrin and metalloproteinase domain 22	3.49	8.96E-05
PLEC1	Plectin 1, intermediate filament binding protein 500kDa	2.86	0.0001
ATP2A2	Sarcoplasmic/endoplasmic reticulum calcium ATPase 2	2.37	0.035
ACTBL2	Beta-actin-like protein 2	2.28	0.008
CTNNB1	Catenin (Cadherin-associated protein), beta 1, 88kDa	2.07	0.02
CFL2	Cofilin-2	2.11	0.0004
CFL1	Cofilin-1 (Non-muscle), isoform	2.01	0.04
DYNC1H1	Cytoplasmic dynein 1 heavy chain 1	1.91	0.04
SRRM2	Serine/arginine repetitive matrix protein 2	1.85	0.006
AMOT	Angiomotin	1.79	0.0005
MYH15	Myosin-15	1.76	0.0003
MYH10	Myosin-10	1.73	0.02
TUBA4B	Putative tubulin-like protein alpha-4B	1.67	0.003
USMG5	Up-regulated during skeletal muscle growth protein 5	1.58	0.02
KIF5C	Kinesin heavy chain isoform 5C	1.58	0.009
TUBA1A	Tubulin alpha-1A chain	1.50	0.01
**Protein Synthesis and Transport, Glycosylation and Glycosaminoglycan Synthesis (GAGs)**			
EIF3C	Eukaryotic translation initiation factor 3 subunit C	7.24	0.0002
VCP	Valosin-containing protein	4.95	7.38E-06
RPL9	60S ribosomal protein L9	4.94	6.46E-05
SARS	Seryl (serine)-tRNA synthetase	4.85	0.0002
PMM2	Phosphomannomutase 2	4.45	1.43E-05
SURF4	Surfeit locus protein 4	3.53	5.63E-05
EEF1G	Elongation factor 1-gamma	3.18	7.42E-05
EIF3A	Eukaryotic translation initiation factor 3 subunit A	3.04	0.0007
RPB4	60S ribosomal protein L4	2.81	0.02
EIF3G	Eukaryotic translation initiation factor 3 subunit G	2.77	7.33E-06
RAB2	RAB2, member RAS oncogene family	2.59	0.03
COPG1	Coatomer subunit gamma-1	2.34	0.003
HSP90AB1	Heat shock protein 90kDa alpha (Cytosolic), class B member 1	2.29	0.02
UGDH	UDP-glucose 6-dehydrogenase	2.28	0.03
EEF1A1	Eukaryotic translation elongation factor 1 alpha 1	2.21	0.004
RRBP1	p180/ribosome receptor	2.19	0.04
AP1G1	AP-1 complex subunit gamma-1	1.98	0.002
RPS18	40S ribosomal protein S18	1.97	0.007
RAB21	RAB21, member RAS oncogene family	1.88	0.02
RPL15	60S ribosomal protein L15	1.82	0.026
RPL7	60S ribosomal protein L7	1.75	0.03
STIP1	Stress-induced-phosphoprotein 1 (Hsp70/Hsp90-organizing protein)	1.72	0.003
P4HB	Protein disulfide-isomerase	1.69	0.02
RPS16	40S ribosomal protein S16	1.59	0.015
RPS2	40S ribosomal protein S2	1.57	0.008
RPL35	60S ribosomal protein L35	1.55	0.02
SEC22B	Vesicle-trafficking protein SEC22b	1.50	0.037
**DNA-binding, Nuclear-related, and Cell Cycle Control**			
PRKDC	DNA-dependent protein kinase catalytic subunit (non-homologous end joining (NHEJ))	14.71	0.02
XRN2	5′-3′ exoribonuclease 2	5.53	0.0006
NAP1L4	Nucleosome assembly protein 1-like 4	4.46	0.002
ESF1	Nucleolar Pre-RRNA Processing Protein, Homolog	4.30	2.00E-08
NASP	Nuclear autoantigenic sperm protein	4.12	0.009
HNRNPA1	Heterogeneous nuclear ribonucleoprotein A1	3.85	9.30E-05
FUBP1	Far upstream element-binding protein 1	3.21	1.44E-05
POLD3	DNA polymerase delta subunit 3	3.09	0.0007
RPA1	Replication protein A 70 kDa DNA-binding subunit	2.80	0.02
CHD4	Chromodomain-helicase-DNA-binding protein 4	2.62	5.68E-05
CTPS1	CTP synthase	2.37	0.0003
LMNA	Prelamin-A/C	2.22	0.01
CEP110	Centrosomal protein 110kDa	2.02	0.03
MCM7	MCM7 minichromosome maintenance deficient 7	1.96	0.03
NAP1L1	Nucleosome assembly protein 1-like 1	1.86	0.015
HDAC1	Histone deacetylase 1	1.71	0.045
SP100	Nuclear autoantigen Sp-100	1.64	0.03
DHX9	ATP-dependent RNA helicase A	1.47	0.02
**Inflammation/Immune Function**			
LTA4H	Leukotriene A(4) hydrolase	3.52	1.16E-06
CCT4	T-complex protein 1 subunit delta	2.13	0.0003
CCT2	T-complex protein 1 subunit beta	1.53	0.04
**Protein Degradation**			
UBR4	E3 ubiquitin-protein ligase UBR4	3.47	0.002
STUB1	E3 ubiquitin-protein ligase CHIP	2.55	0.01
PSMC3	Proteasome (prosome, macropain) 26S subunit, ATPase 3	1.79	0.04
PSMB4	Proteasome subunit beta type-4	1.72	0.003
PSMD2	Proteasome 26S non-ATPase subunit 2	1.58	0.04
UBE1	Ubiquitin-activating enzyme E1 (A1S9T and BN75 temperature sensitivity complementing)	1.55	0.003
UBE2V1	Ubiquitin-conjugating enzyme E2 variant 1	1.47	0.03
USP14	Ubiquitin carboxyl-terminal hydrolase 14	1.47	0.007

Note that doxycycline targets mitochondrial metabolism, glycolysis, the EMT, protein synthesis and the DNA damage response, as well as inflammation and protein degradation, in human breast cancer cells.

Interestingly, using this approach, we identified DNA-PK as the protein target that was most dramatically down-regulated by doxycycline, by nearly 15-fold (> 90% reduction) (Table 1). DNA-PK (also known as PRKDC) is the catalytic subunit of the DNA-dependent protein kinase involved in DNA-repair. DNA-PK is required for DNA-repair using the mechanism of NHEJ (non-homologous end joining) [18] [19]. DNA-PK also functions to maintain the integrity and copy number of mt-DNA, so there is a clear link with mitochondrial metabolic function, as well [20].

Consistent with our current findings, we also observed that DNA-PK is significantly over-expressed in both MCF7 and T47D mammospheres (Table 2). Remarkably, DNA-PK is infinitely upregulated in MCF7 mammospheres and nearly 15-fold increased in T47D mammospheres, relative to monolayer cultures.

**Table 2:** DNA-PK is highly up-regulated in both MCF7 and T47D mammospheres, as compared with monolayer cultures

Cell Line	Symbol	Gene Description	Up-regulation (fold-change)	ANOVA
MCF7	PRKDC	DNA-dependent protein kinase, catalytic subunit	Infinity	1.13E-10
T47D	PRKDC	DNA-dependent protein kinase, catalytic subunit	14.85	2.60E-05

### Clinical relevance of “doxycycline reduced targets” in human breast cancers

To determine the potential clinical relevance of our proteomic findings, we next assessed whether the protein targets reduced by doxycycline were transcriptionally upregulated in human breast cancer cells *in vivo*.

For this purpose, we employed a published clinical data set of *N* = 28 breast cancer patients in which their tumor samples were subjected to laser-capture micro-dissection, to physically separate epithelial cancer cells from their adjacent tumor stroma [21]. Table 3 presents a summary of these findings. Importantly, most of the protein targets reduced by doxycycline were transcriptionally upregulated in human breast cancer cells in tumors excised from patients.

**Table 3:** Doxycycline-targets normally up-regulated in human breast cancer cells *in vivo*

Symbol	Gene Description	Up-regulation (fold-change)	*P*-value
**Mitochondrial-related Proteins**			
ATP5F1	ATP synthase, H+ transporting, mitochondrial F0 complex, subunit b	5.39	7.83E-07
COX5B	Cytochrome c oxidase subunit 5B, mitochondrial	5.03	2.86E-06
UQCRC2	Cytochrome b-c1 complex subunit 2, mitochondrial	4.84	5.73E-06
COX6A	Cytochrome c oxidase subunit 6A, mitochondrial	4.46	2.07E-05
LRPPRC	Leucine-rich PPR-motif containing (inhibitor of mitophagy)	4.34	3.15E-05
NDUFA4	NADH dehydrogenase (Ubiquinone) 1 alpha-subcomplex, 4	4.25	4.29E-05
MDH2	Malate dehydrogenase 2, mitochondrial	4.18	5.32E-05
HSPD1	60 kDa heat shock protein, mitochondrial	3.42	5.93E-04
DECR1	2,4-dienoyl CoA reductase 1, mitochondrial	3.38	6.86E-04
VDAC1	Voltage-dependent anion-selective channel protein 1	2.64	5.35E-03
PRKDC	DNA-dependent protein kinase catalytic subunit (maintains mt-DNA integrity & copy number)	2.14	0.02
**Glycolytic Enzymes**			
TPI1	Triosephosphate isomerase 1	4.21	4.88E-05
ALDOA	Fructose-bisphosphate aldolase A	3.60	3.45E-04
GPI	Glucose-6-phosphate isomerase	3.36	7.28E-04
PKM2	Pyruvate kinase	3.26	9.79E-04
PGK1	Phosphoglycerate kinase 1	2.46	8.66E-03
LDHA	L-lactate dehydrogenase A	2.42	9.42E-03
ENO1	Enolase 1	1.96	0.03
**EMT-markers/Muscle-related proteins (cytoskeletal)**			
CFL1	Cofilin-1 (Non-muscle), isoform	2.39	0.01
TUBB1	Tubulin beta-1 chain	2.32	0.01
TUBA1A	Tubulin alpha-1A chain	2.17	0.02
ATP2A2	Sarcoplasmic/endoplasmic reticulum calcium ATPase 2	2.07	0.02
CTNNB1	Catenin (Cadherin-associated protein), beta 1, 88kDa	2.05	0.02
MYH10	Myosin-10	1.82	0.037
KIF5C	Kinesin heavy chain isoform 5C	1.82	0.037
**Protein Synthesis and Transport**			
RPL7	60S ribosomal protein L7	5.21	1.53E-06
RPS18	40S ribosomal protein S18	4.96	3.71E-06
HSP90AB1	Heat shock protein 90kDa alpha (Cytosolic), class B member 1	4.94	4.03E-06
RPS2	40S ribosomal protein S2	4.77	7.21E-06
RPL15	60S ribosomal protein L15	4.60	1.28E-05
EIF3C	Eukaryotic translation initiation factor 3 subunit C	4.48	1.94E-05
RPL35	60S ribosomal protein L35	4.10	7.05E-05
RAB2	RAB2, member RAS oncogene family	3.73	2.29E-04
RPL9	60S ribosomal protein L9	3.71	2.49E-04
EEF1G	Elongation factor 1-gamma	3.71	2.44E-04
EEF1A1	Eukaryotic translation elongation factor 1 alpha 1	3.16	1.30E-03
RPS16	40S ribosomal protein S16	3.05	1.77E-03
RPL4	60S ribosomal protein L4	3.05	1.79E-03
SEC22B	Vesicle-trafficking protein SEC22b	3.04	1.81E-03
EIF3A	Eukaryotic translation initiation factor 3 subunit A	2.51	7.57E-03
SARS	Seryl (serine)-tRNA synthetase	2.15	0.02
P4HB	Protein disulfide-isomerase	2.15	0.02
EIF3G	Eukaryotic translation initiation factor 3 subunit G	1.92	0.03
**DNA-binding, Nuclear-related, and Cell cycle control**			
HDAC1	Histone deacetylase 1	4.15	6.02E-05
NAP1L4	Nucleosome assembly protein 1-like 4	4.07	7.61E-05
HNRNPA1	Heterogeneous nuclear ribonucleoprotein A1	4.03	8.90E-05
RPA1	Replication protein A 70 kDa DNA-binding subunit	2.52	7.30E-03
MCM7	MCM7 minichromosome maintenance deficient 7	2.23	0.015
NAP1L1	Nucleosome assembly protein 1-like 1	2.19	0.017
PRKDC	DNA-dependent protein kinase catalytic subunit (non-homologous end joining (NHEJ))	2.14	0.02
CTPS1	CTP synthase	2.09	0.02
CHD4	Chromodomain-helicase-DNA-binding protein 4	1.87	0.034
**Inflammation/Immune Function**			
CCT4	T-complex protein 1 subunit delta	3.25	1.00E-03
LTA4H	Leukotriene A(4) hydrolase	2.94	2.40E-03
CCT2	T-complex protein 1 subunit beta	2.85	3.06E-03
**Protein Degradation**			
PSMB4	Proteasome subunit beta type-4	4.16	5.70E-05
PSMD2	Proteasome 26S non-ATPase subunit 2	3.31	8.29E-04
USP14	Ubiquitin carboxyl-terminal hydrolase 14	3.23	1.05E-03
STUB1	E3 ubiquitin-protein ligase CHIP	1.95	0.03
UBR4	E3 ubiquitin-protein ligase UBR4	1.77	0.04

In light of these findings, the new “doxycycline reduced targets” that we identified may be particularly relevant, for improving both the diagnosis and treatment of human breast cancers.

### Validation of DNA-PK as a therapeutic target in CSCs

**Figure 4:**
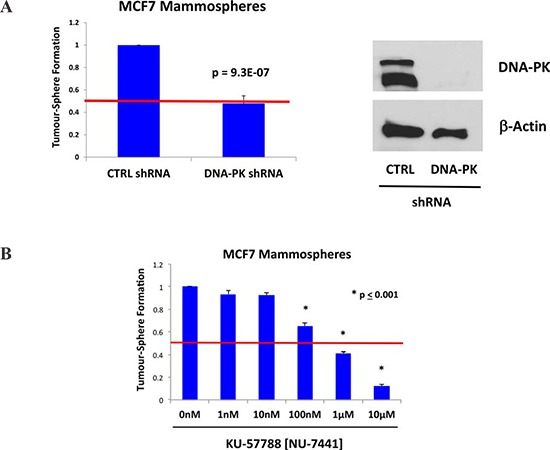
DNA-PK is required for mammosphere formation in MCF7 cells **A.** Genetic approach. MCF7 cells were transduced with lentiviral vectors harboring either control sh-RNA or sh-RNA species targeting the expression of DNA-PK. Note that MCF7 cells harboring the DNA-PK sh-RNA show a 50% reduction in mammosphere forming capacity, as predicted. **B.** Pharmacological approach. Note that the well-established DNA-PK inhibitor, namely KU-57788 [NU-7441], dose-dependently inhibits MCF7 mammosphere formations, with an IC-50 between 100 nM and 1 μM. As such, DNA-PK activity is required for the efficient clonal expansion and anchorage-independent growth of CSCs, as observed using the mammosphere assay. Each data point in this figure is the average of 9 replicates.

To validate the potential relevance of DNA-PK for maintaining the growth of CSCs, we next used an sh-RNA-approach. Briefly, MCF7 cells were transduced with lentiviral vectors harboring either control sh-RNA or sh-RNA species targeting the expression of DNA-PK. Figure 4A illustrates that MCF7 cells harboring the DNA-PK sh-RNA show a 50% reduction in mammosphere forming capacity, as predicted. Loss of DNA-PK expression was indeed confirmed by immunoblot analysis, using specific antibody probes (Figure 4A).

Validation was also performed using a well-established DNA-PK inhibitor, namely KU-57788 [NU-7441] [22, 23]. Figure 4B directly shows that KU-57788 [NU-7441] dose-dependently inhibits MCF7 mammosphere formations, with an IC-50 between 100 nM and 1 μM.

Thus, DNA-PK activity appears to be required for the efficient clonal expansion and anchorage-independent growth of CSCs, as observed using the mammosphere assay.

### Doxycycline pre-treatment sensitizes CSCs to radiation treatment

Since DNA-PK has been previously implicated in the radio-resistance of cancer cells [24, 25] and we show here that doxycycline functionally reduces the expression of DNA-PK, we would predict that doxycycline treatment should radio-sensitize CSCs.

**Figure 5:**
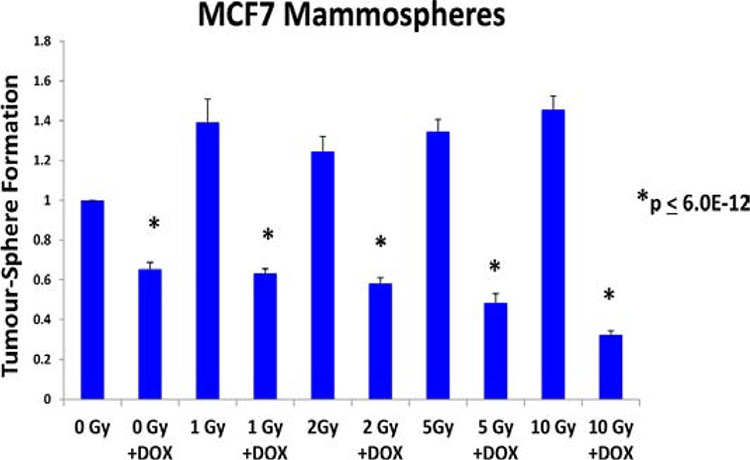
Doxycycline pre-treatment sensitizes cancer stem cells to radiation treatment MCF7 cell monolayers were pre-treated with doxycycline (50 μM) for 3-days and then irradiated. After radiation treatment, monolayers were trypsinized and re-plated to evaluate mammosphere growth over a 5-day period. Note that radiation treatment significantly increases the growth of CSCs by up to 1.45-fold, as expected. In contrast, doxycycline pre-treatment increased the sensitivity of CSCs to radiation by up to 4.5-fold. In conclusion, doxycycline pre-treatment functionally sensitizes CSCs to radiation, as predicted based on its ability to reduce DNA-PK expression. Each data point in this experiment is the average of 18 replicates.

**Figure 6:**
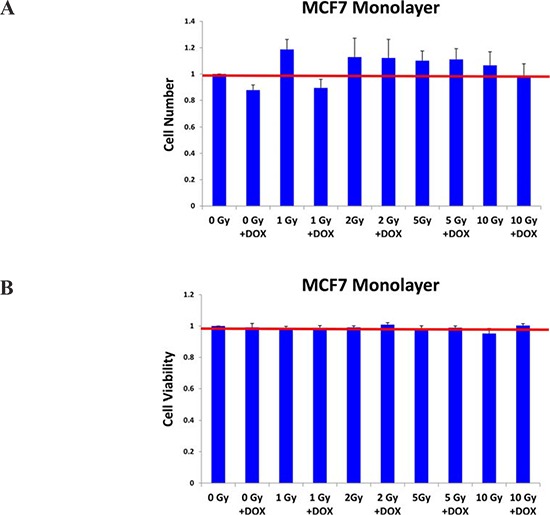
Doxycycline pre-treatment does not inhibit the growth and viability of MCF7 cell monolayers As in Figure 4, MCF7 cell monolayers were pre-treated with doxycycline (50 μM) for 3-days and then irradiated. However, note that under these conditions doxycycline pre-treatment (with or without radiation), had little or no effect on the proliferation or viability of the “bulk” cancer cells. Each data point in this experiment is the average of 18 replicates.

To test this hypothesis directly, MCF7 cell monolayers were pre-treated with doxycycline (50 μM) for 3-days and then irradiated. After radiation treatment, monolayers were trypsinized and re-plated to evaluate mammosphere growth over a 5-day period. Figure 5 shows that radiation treatment significantly increases the growth of CSCs by up to 1.45-fold, as expected. In contrast, doxycycline pre-treatment increased the sensitivity of CSCs to radiation by up to 4.5-fold. However, under these conditions, doxycycline pre-treatment (with or without radiation) had little or no effect on the proliferation or viability of the “bulk” cancer cells (Figure 6A, 6B).

Thus, doxycycline pre-treatment functionally sensitizes CSCs to radiation, as predicted based on its ability to reduce DNA-PK expression.

### Validation of the metabolic phenotype induced by doxycycline pre-treatment

Based on our proteomics analysis presented in Table 1, both the levels of key mitochondrial proteins and glycolytic enzymes were significantly reduced by doxycycline pre-treatment. Thus, these results suggest that doxycycline should reduce overall metabolic activity in cancer cells.

**Figure 7:**
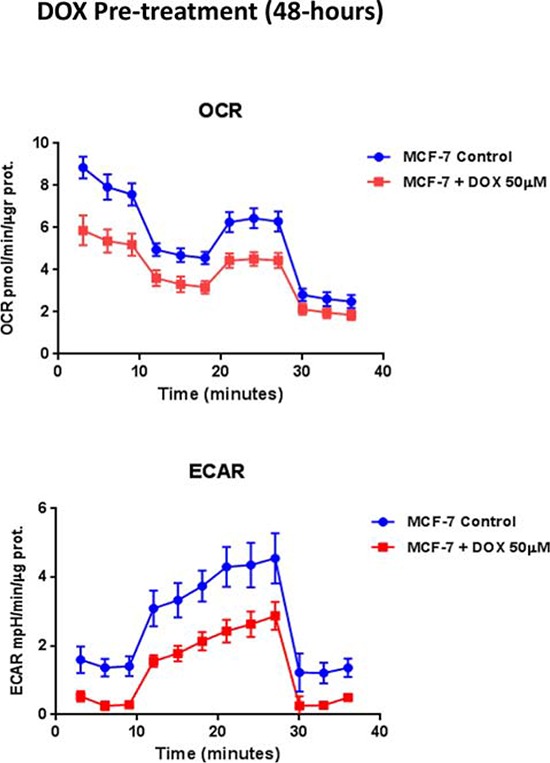
Doxycycline treatment reduces the rates of both oxidative mitochondrial metabolism and glycolysis We examined the metabolic profile of MCF7 cell monolayers pre-treated with doxycycline (50 μM) for 2-days. Note that the rates of both oxidative mitochondrial metabolism and glycolysis were significantly reduced by doxycycline pre-treatment, as measured using the Seahorse XFe96 analyzer to measure metabolic flux.

**Figure 8:**
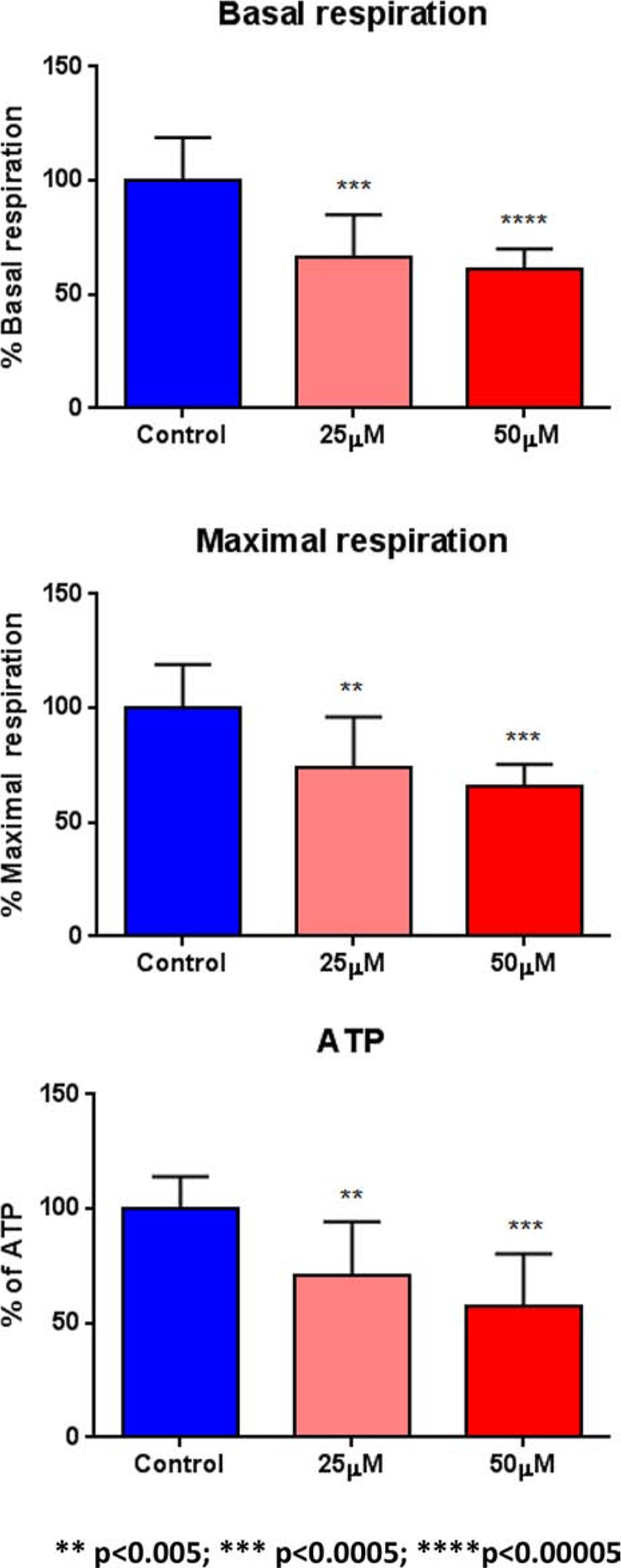
Doxycycline quantitatively reduces respiration (basal and maximal) and ATP levels We examined the metabolic profile of MCF7 cell monolayers pre-treated with doxycycline (50 μM) for 2-days, using the Seahorse XFe96 analyzer to measure metabolic flux. Note that significant reductions in respiration (basal and maximal), as well as reduced ATP levels, were observed experimentally. Each data point in this figure is the average of 9 replicates.

**Figure 9:**
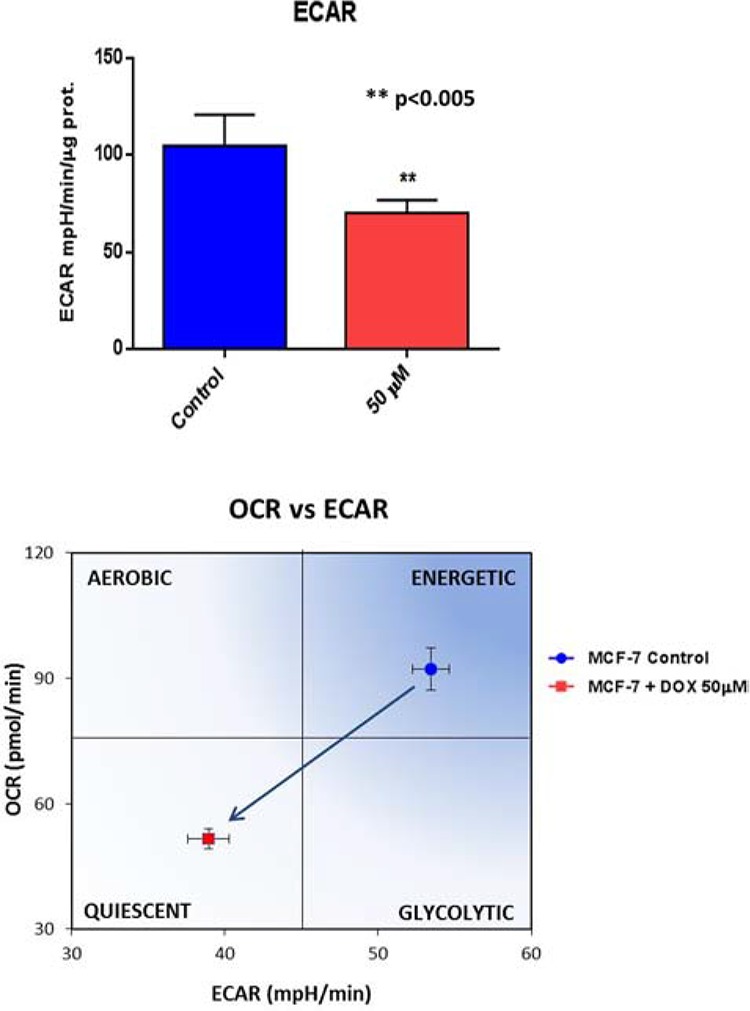
Doxycycline shifts MCF7 cancer cells from a highly energetic to a metabolically quiescent state We examined the metabolic profile of MCF7 cell monolayers pre-treated with doxycycline (50 μM) for 2-days, using the Seahorse XFe96 analyzer to measure metabolic flux. Note that MCF7 cancer cells were shifted towards a metabolically quiescent state. Each data point in this figure is the average of 9 replicates.

To test this hypothesis further, we next examined the metabolic profile of MCF7 cell monolayers pre-treated with doxycycline (50 μM) for 2-days. Interestingly, Figure 7 shows that the rates of both oxidative mitochondrial metabolism and glycolysis were dramatically reduced by doxycycline pre-treatment, as measured using the Seahorse XFe96 analyzer to measure metabolic flux. This resulted in significant reductions in respiration (basal and maximal), as well as reduced ATP levels (Figure 8). Finally, as seen in Figure 9, MCF7 cancer cells were shifted from a highly energetic to a metabolically quiescent state.

### Doxycycline reduces the anoikis-resistance of MCF7 cells, prior to mammosphere formation

Anoikis is a specific type of programmed cell death (apoptosis) that is induced in certain cell types, under anchorage-independent growth conditions [26, 27]. Interestingly, epithelial CSCs show anoikis-resistance, and are able to undergo mammosphere formation under these anchorage-independent conditions [28].

Next, to determine the possible effects on anoikis-resistance, MCF7 cells were pre-treated with doxycycline (at 25 or 50 μM) as monolayers for 2-days and then re-plated on low-attachment plates, for either the anoikis assay or the mammosphere assay, in the absence of doxycycline.

**Figure 10:**
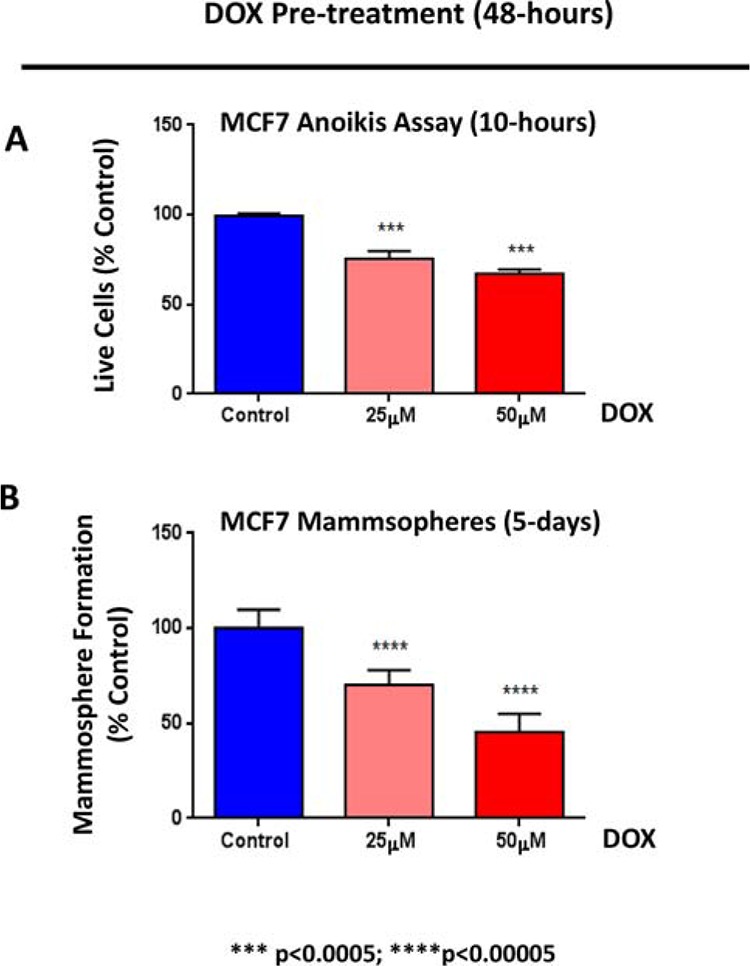
Doxycycline reduces the anoikis-resistance of MCF7 cells, prior to mammosphere formation MCF7 cells were pre-treated with doxycycline (at 25 or 50 μM) as monolayers for 2-days and then re-plated on low-attachment plates, for the anoikis assay or the mammosphere assay, in the absence of doxycycline. **A.** *Anoikis Assay*. Note that doxycycline pre-treatment dose-dependently reduced the number of live cells remaining after 10 hours of seeding on low-attachment plates. **B.** *Mammosphere formation*. Note that, under these conditions, doxycycline pre-treatment dose-dependently reduced the mammosphere forming capacity of MCF7 cells, by up to ~ 50%. Each data point in this figure is the average of 9 replicates.

Importantly, Figure 10 shows that doxycycline pre-treatment dose-dependently reduced the number of live cells remaining after 10 hours of seeding on low-attachment plates. Under these conditions, doxycycline pre-treatment also dose-dependently reduced the mammosphere forming capacity of MCF7 cells, by up to ~ 50%. Thus, doxycycline may exert its inhibitory effects on mammosphere formation, in part, by effectively reducing anoikis-resistance, during the initial time spent under low-attachment conditions.

### Doxycycline simultaneously inhibits the functional activity of multiple stem-cell associated signal transduction pathways

To better understand which signaling pathways are affected by doxycycline treatment, we used a panel of eight MCF7-GFP cell lines engineered to express different luciferase-based transcriptional reporters. The functional activation state of these reporters was then normalized by GFP expression, to account for cell number.

**Figure 11:**
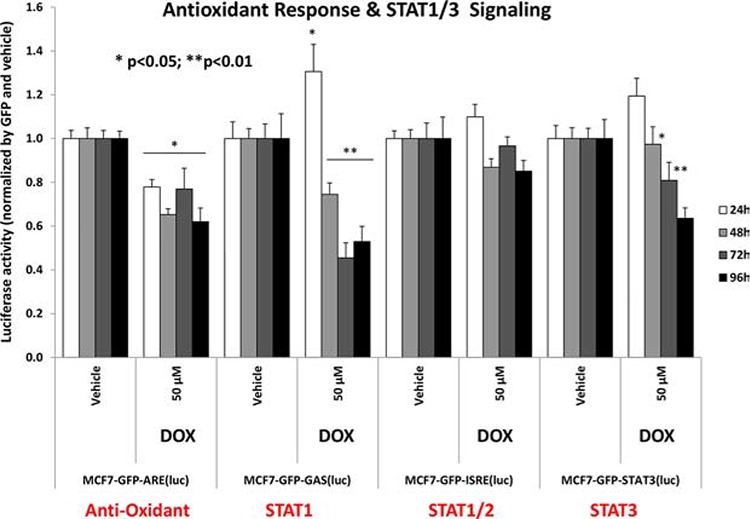
Doxycycline treatment of MCF7 monolayers inhibits the anti-oxidant response (NRF1/2) and STAT1/3 signaling Note that doxycycline is especially effective at 72 and 96 hours. Each data point in this figure is the average of at least 8 replicates.

**Figure 12:**
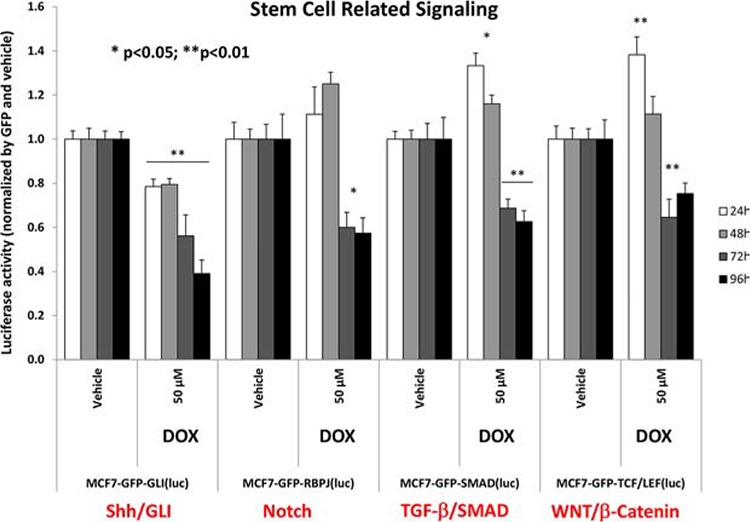
Doxycycline dampens signaling along four major stem-cell associated pathways in MCF7 cells, namely Sonic Hedgehog, Notch, WNT and TGF-beta signaling Note that the functional effects of doxycycline were most prominent at 72 and 96 hours. In several cases, a bi-phasic response was noted, with activation of signaling at 24 hours, and progressive inhibition from 48-to-96 hours. Each data point in this figure is the average of at least 8 replicates.

Interestingly, Figure 11 directly shows that doxycycline treatment of MCF7 monolayer cultures inhibits both the anti-oxidant response (NRF1/2) and STAT1/3 signaling, especially at 72 and 96 hours. Similarly, Figure 12 illustrates that doxycycline also dampens signaling along four other stem-cell associated pathways, including Sonic Hedgehog, Notch, WNT and TGF-beta signaling, again most prominently at 72 and 96 hours. Interestingly, in several cases, a bi-phasic response was noted, with activation of signaling at 24 hours, and progressive inhibition from 48-to-96 hours.

These results directly support our observation that doxycycline potently inhibits mammosphere formation, by blocking the clonal expansion of CSCs and reduces anoikis-resistance. Indeed, it has been reported that most of these signaling pathways such as Notch, Hedgehog, WNT, TGFβ, STAT3 or NRF1/2 are able to confer anoikis-resistance, and inhibition of these pathways sensitizes CSCs to anoikis.

## DISCUSSION

Recently, we proposed that FDA-approved antibiotics that adversely effect mitochondria could be used to effectively target the cancer stem cell population, by inhibiting mitochondrial biogenesis in tumor-initiating cells (TICs) [7]. One of these promising antibiotics is doxycycline, a member of the tetracycline class, with excellent pharmacokinetics.

To better understand how doxycycline exerts its therapeutic effects, here we used a chemical proteomics approach [29, 30] to more effectively interrogate the target-phenotype relationship. Our results directly show that doxycycline treatment significantly reduced the expression of many key protein targets functionally associated with mitochondrial metabolism, glycolysis, the EMT, protein synthesis and the DNA damage response, as well as inflammation and protein degradation, in human breast cancer cells. We further validated that doxycycline suppresses both oxidative mitochondrial metabolism and glycolysis, using metabolic flux analysis. Doxycycline also inhibited signal transduction along multiple stem-cell associated communication pathways, namely Sonic Hedgehog, Notch, WNT and TGF-beta signaling.

**Figure 13:**
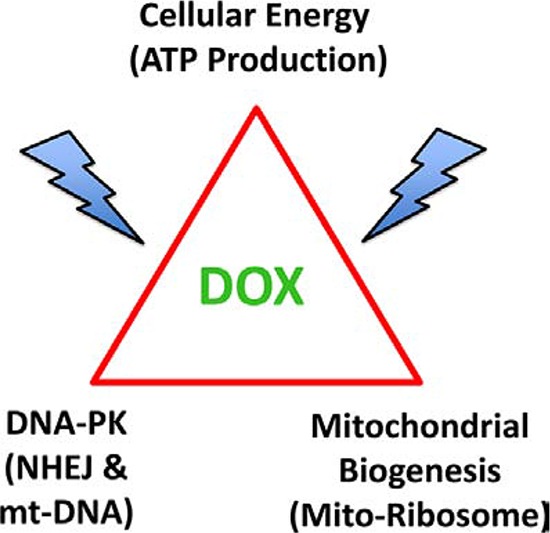
Doxycycline targets mitochondrial biogenesis and DNA-repair, ultimately converging on ATP production and energy metabolism in cancer cells DNA-PK activity is normally required for repairing mt-DNA and maintaining mt-DNA copy number. Mitochondrial biogenesis is dependent on mitochondrial protein synthesis, which is carried out, in part, by mitochondrial ribosomes, which share significant homology with bacterial ribosomes. Doxycycline targets both mitochondrial ribosomes and DNA-PK, thereby reducing ATP levels, as observed experimentally.

Interestingly, using this approach, we also identified DNA-PK as the protein target that was most dramatically down-regulated by doxycycline, by nearly 15-fold (> 90% reduction) (Figure 13). DNA-PK is required for effective cellular DNA-repair and also maintains the integrity and copy number of mitochondrial DNA [20, 31, 32]. DNA-PK is thought to confer radiation resistance in cancer cells [33–35], but has never been previously implicated in the growth of CSCs.

Additional validation experiments, using both genetic (DNA-PK sh-RNA) and pharmacological (DNA-PK inhibitor: NU-7441) approaches directly showed that DNA-PK expression or activity is required for MCF7 cells to efficiently form mammospheres. This is the first demonstration that DNA-PK is required for the clonal expansion and survival of CSCs. Most importantly, however, pre-treatment of MCF7 cell monolayers with doxycycline was sufficient to increase the sensitivity of CSCs to radiation treatment, by up to 4.5-fold.

Unfortunately, no FDA-approved DNA-PK inhibitors have emerged, despite many years of drug discovery and lead optimization. This is largely because existing DNA-PK inhibitors suffer from poor pharmacokinetics. They are not well absorbed and/or are unstable, with a short plasma half-life [36–38]. In contrast, doxycycline has excellent pharmacokinetics, with nearly 100% oral absorption and a long serum half-life (18–22 hours), at a standard dose of 200-mg per day [39, 40].

Thus, we propose that the efficacy of doxycycline as a DNA-PK inhibitor should be tested in Phase-II clinical trials, in combination with radio-therapy. In further support of this idea, we show that doxycycline effectively inhibits the mammosphere-forming activity of primary breast cancer samples, derived from metastatic disease sites (pleural effusions or ascites fluid).

## MATERIALS AND METHODS

### Materials

Breast cancer cell lines (MCF7 and T47D) were purchased from the ATCC. Gibco-brand cell culture media (DMEM and DMEM/F12) was purchased from Life Technologies. Doxycycline was purchased from Sigma-Aldrich. Sh-RNA lentiviral particles targeting DNA-PK (sc-35200-V) were obtained commercially from Santa Cruz Biotech (USA), along with appropriate sh-RNA control particles (sc-108080). Antibodies directed against DNA-PK (MS-423-P0) for immunoblot analysis were obtained from Thermo Scientific. KU-57788 [NU-7441] was obtained from Selleckchem.

### Description of primary tumor samples from metastatic disease sites

Ethical approval was granted by the Central Office for Research Ethics Committee (study numbers: 05/Q1402/25 and 05/Q1403/159) and patients gave written informed consent. Metastatic fluid samples were obtained from 4 patients undergoing palliative drainage of symptomatic ascites or pleural effusions at The Christie Hospital, Manchester, UK. Estrogen, progesterone, and HER2 receptor status of the primary tumors were reported by the Department of Pathology at The Christie, according to established criteria [41].

### Isolation of breast cancer epithelial cells

Metastatic breast cancer cells were harvested as previously described, with minor modifications [42]. Invasive breast cancer tissue (1–2 cm^3^) was collected, dissected into 1–2 mm^3^ cubes, and digested in media comprised of Dulbecco's Modified Eagle's Media (DMEM), 15 mM HEPES, and 10% collagenase/hyaluronidase (Stem Cell Technologies) (supplemented with Pen-Strep) at 37°C for 16 hours. Digested tissue was filtered sequentially through 100, 70, and 40 μm sieves. Red blood cells were removed using Lymphoprep (Axis-Shield), and leukocytes were removed with CD45-negative magnetic sorting according to the manufacturer's instructions (Miltenyi Biotech).

### Pre-treatment of monolayers with doxycycline, with and without radiation treatment

MCF7 cells were plated in normal medium (DMEM, 10% FCS, L-glutamine, supplemented with Pen-Strep) for 24-hr. Cells were then treated, initially for 7 days with doxycycline, with media changed every 3 days. For subsequent experiments, cells were treated for 72-hr. For radiation experiments, monolayer cells pre-treated with doxycycline, were exposed to increasing levels of radiation, and the cells collected by trypsinization and centrifugation. To quantitatively determine cell growth, the number of cells remaining after treatment was counted using an automatic cell counter (Biorad), compared to untreated cells and expressed as fold-change. To assess cell viability, cells were incubated for 1 minute with Trypan Blue (Sigma, #T8145) using a 1:1 ratio. The number of Trypan Blue positive cells (non-viable) was measured using an automatic cell counter (Biorad) and compared to untreated controls. Cells were also plated into mammosphere cultures to assess stem cell-like activity with no further drug treatment. All experiments were performed in triplicate and repeated at least three times independently.

### Irradiation of MCF7 monolayers using a cell x-ray unit

MCF7 cells were pre-treated at a monolayer for 3 days, with either doxycycline (50 μM) or with vehicle-alone, and then were irradiated with 0–10 Gy at room temperature, using an X-ray unit, at a dose-rate of 1.37 Gy/min. More specifically, irradiation was performed using a free-standing 320 kV x-ray system (Gulmay Medical Ltd, now Xstrahl Ltd, Camberley, UK). The machine was operated at 300 kV, 10 mA with the addition of a 0.75 mm Cu filter to give a beam quality with half-value layer (HVL) of 2.3 mm Cu. Samples were positioned at a distance of 500 mm from the x-ray focus and a backscatter block was utilised to ensure good dose uniformity. The dose rate to samples was determined to be 1.37 Gy/min [43]. This dose rate was checked regularly using the IPEM 2005 protocol and equipment whose calibration was traceable to UK national standards.

### Lentiviral transduction

Lentiviral particles harboring human DNA-PK sh-RNA (#sc-35200-V, Santa Cruz) or control shRNA lentiviral Particles (#sc-108080, Santa Cruz), were used to stably transduce MCF7 cells, according to the manufacturer's protocol (in the presence of 5 μg/ml polybrene). Twenty-four hours post-infection, media containing the virus was removed and replaced with standard media. Cells were then selected with 2 μg/ml puromycin, for up to 10 days.

### Immunoblot analysis

MCF7 cells were seeded in 10 cm dishes for 72 hrs. Then, cells were lysed in RIPA buffer (Sigma), containing proteinase inhibitors (Roche) and kept at 4°C for 30 minutes. Lysates were collected by centrifugation for 10 minutes at 10,000 × g, and protein concentration were determined using the BCA protein assay kit (Pierce). Samples were diluted into SDS-PAGE sample buffer and were boiled for 5 minutes before being separated by SDS-PAGE, using a 4–15% gradient Mini-PROTEAN TGX Gel (Biorad). Samples were then transferred onto a nitrocellulose membrane (Biorad), blocked in 5% milk in TBS-Tween 20 (Sigma) and probed with antibodies directed against DNA-PK (ThermoScientific, #MS-423-P0) and β-actin (Santa Cruz Biotechnology, #sc-1616), using a secondary antibody at a dilution of 1 to 5000. Bound antibodies were detected using the Supersignal West Pico Chemiluminiscent substrate (ThermoScientific).

### Mammosphere culture

A single cell suspension was prepared using enzymatic (1x Trypsin-EDTA, Sigma Aldrich, #T3924), and manual disaggregation (25 gauge needle) to create a single cell suspension [44]. Cells were plated at a density of 500 cells/cm2 in mammosphere medium (DMEM-F12/B27/20 ng/ml EGF/PenStrep) in non-adherent conditions, in culture dishes coated with (2-hydroxyethylmethacrylate) (poly-HEMA, Sigma, #P3932). Cells were grown for 5 days and maintained in a humidified incubator at 37°C at an atmospheric pressure in 5% (v/v) carbon dioxide/air. After 5 days for culture, spheres > 50 μm were counted using an eye piece graticule, and the percentage of cells plated which formed spheres was calculated and is referred to as percentage mammosphere formation, and was normalized to one (1 = 100% MSF). For proteomic analysis, mammospheres were collected by centrifugation at 800 rpm for 10 minutes and compared to monolayer cells grown for 5 days.

### Label-free quantitative proteomics analysis

Cell lysates were prepared for trypsin digestion by sequential reduction of disulphide bonds with TCEP and alkylation with MMTS [45]. Then, the peptides were extracted and prepared for LC-MS/MS. All LC-MS/MS analyses were performed on an LTQ Orbitrap XL mass spectrometer (Thermo Scientific, San Jose, CA) coupled to an Ultimate 3000 RSLCnano system (Thermo Scientific, formerly Dionex, The Netherlands). Xcalibur raw data files acquired on the LTQ-Orbitrap XL were directly imported into Progenesis LCMS software (Waters Corp., Milford, MA, formerly Non-linear dynamics, Newcastle upon Tyne, UK) for peak detection and alignment. Data were analyzed using the Mascot search engine. Five replicates were analyzed for each sample type (*N* = 5). Statistical analyses were performed using ANOVA and only fold-changes in proteins with a *p*-value less than 0.05 were considered significant.

### Data mining

To firmly establish the clinical relevance of our results from the quantitative proteomics analysis of mammosheres, we re-analyzed the transcriptional profiles of epithelial breast cancer cells and adjacent tumor stromal cells that were physically separated by laser-capture microdissection (from *N* = 28 human breast cancer patients) [21].

### Seahorse XFe96 metabolic flux analysis

Extracellular acidification rates (ECAR) and real-time oxygen consumption rates (OCR) for MCF7 and MCF7 treated with doxycycline were determined using the Seahorse Extracellular Flux (XFe96) analyzer (Seahorse Bioscience, MA, USA). MCF7 cells were maintained in DMEM supplemented with 10% FBS (fetal bovine serum), 2 mM GlutaMAX, and 1% Pen-Strep. We seeded 7,000 cells per well into XFe96 well cell culture plates and they were incubated overnight at 37°C in a 5% CO_2_ humidified atmosphere. After 24 h, the cells were treated with DOX (25 μM and 50 μM) for 48 h in XFe96 cell culture plates. After 48 h of incubation in DMEM media, MCF-7 cells were washed in XF assay media (or for OCR measurement, XF assay media supplemented with 10 mM glucose, 1 mM Pyruvate, 2 mM L-glutamine and adjusted at 7.4 pH), which were pre-warmed to 37°C. MCF7 cells were then maintained in 175 μL/well of XF assay media at 37°C, in a non-CO2 incubator for 1 h. During the cell incubation time, we loaded 25 μL of 80 mM glucose, 9 μM oligomycin, 1M 2-deoxyglucose (for ECAR measurement) and 10 μM oligomycin, 9 μM FCCP, 10 μM rotenone, 10 μM antimycin A (for OCR measurement), in XF assay media into the injection ports in the XFe96 sensor cartridge. Measurements were normalized by protein content. Data set was analyzed by XFe96 software and GraphPad Prism software, using one-way ANOVA and Student's *t*-test calculations. All experiments were performed in quintuplicate, three times independently, such that each data point represents the average of 15 replicates.

### Anoikis assay

Following doxycycline treatment, the CSC population was enriched by seeding on low-attachment plates. Under these conditions, the non-CSCs undergo anoikis (a form of apoptosis induced by a lack of cell-substrate attachment) and CSCs are believed to survive. The surviving “CSC fraction” was analyzed by FACS analysis. Briefly, 1 × 10^4^ MCF7 cells were treated with doxycycline (25 μM and 50 μM) for 48 h in 6-well plates, grown as a monolayer. Then, the monolayer cells were trypsinized and seeded in low-attachment plates in mammosphere media. After 10 h under low-attachment conditions, MCF7 cells were spun down and incubated with LIVE/DEAD dye (Fixable Dead Violet reactive dye; Invitrogen) for 20 minutes to distinguish between the live and dead populations of cells (cell viability), during anoikis. Samples were then analyzed by FACS (Fortessa, BD Bioscence) and the data were analysed using FlowJo software.

### Monitoring cell signal transduction pathways

The Cignal Lenti luciferase reporter assay was used to monitor the activity of several signaling pathways in MCF7-GFP cells, essentially as previously described [46, 47]. Briefly, viral particles diluted 1:10 in complete media containing polybrene (sc-134220, Santa Cruz) were added to the cells. Puromycin treatment (P9620, Sigma) was added 48 h later in order to stably select infected cells. Luciferase assays (E1501, Promega) were performed according to manufacturer's instructions. Approximately 6 × 10^3^ MCF7 cells were seeded in black-walled 96 well plates. When cells were attached, drug treatments were added for 24, 48, 72 and 96 h. Four replicates were used for each condition. After treatment, the luciferase assay was performed according to manufacturer's instructions and light signal was acquired in the Xenogen VivoVision IVIS Lumina. Results were normalized by GFP fluorescence.

## SUPPLEMENTARY FIGURE



## Abbreviations

CSCs: cancer stem-like cells
TICs: tumor-initiating cells
