# Supplementary material for: Doxycycline down-regulates DNA-PK and radiosensitizes tumor initiating cells: Implications for more effective radiation therapy
Source: Oncotarget. 2015 Jun 13;6(16):14005–25. doi: 10.18632/oncotarget.4159 (PMC4546447; doi:10.18632/oncotarget.4159)
Supplement: Supplementary file 1 [file oncotarget-06-14005-s001.pdf]

SUPPLEMENTARY FIGURE

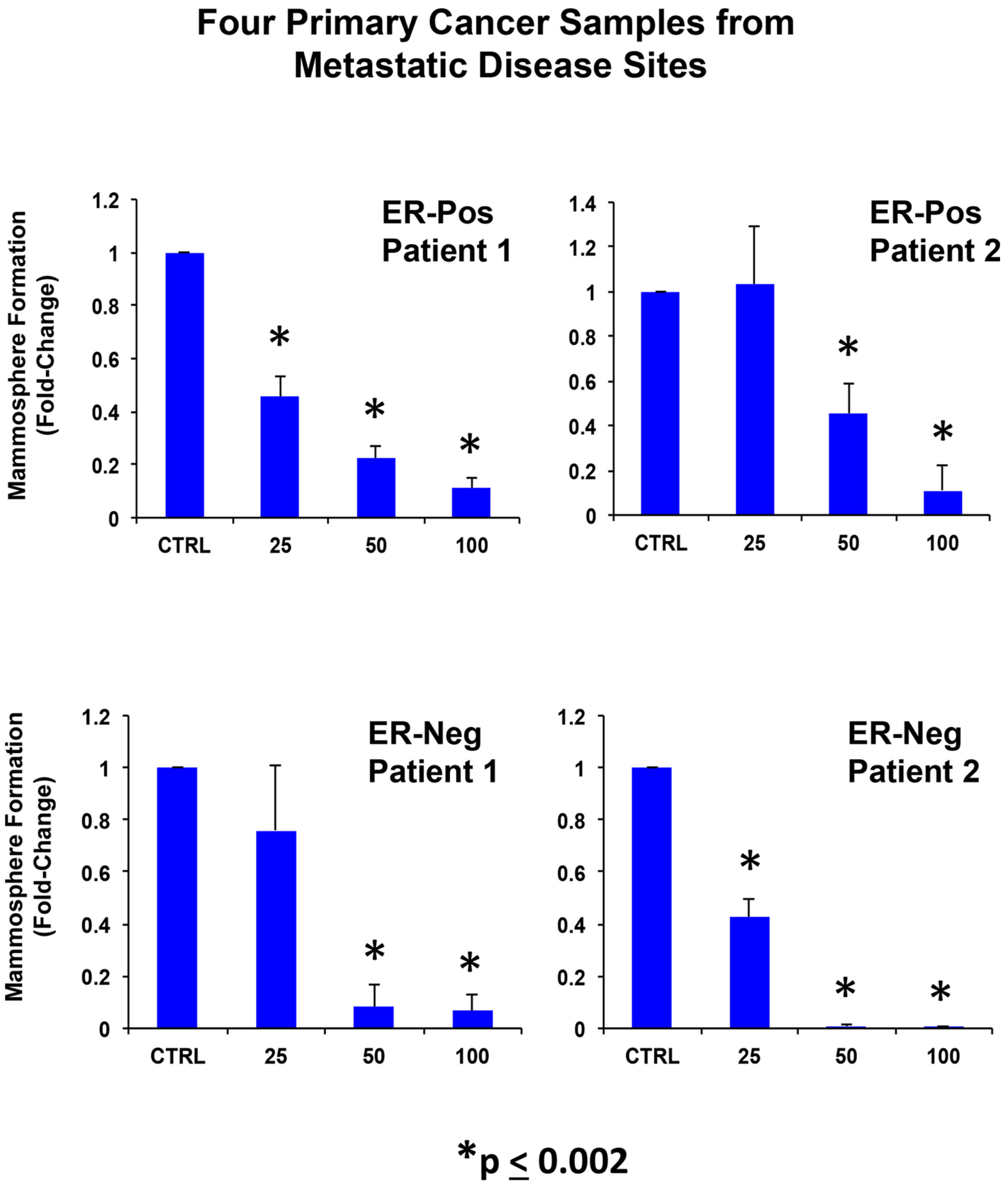

Supplementary Figure S1: Doxycycline inhibits mammosphere formation, as assessed using four primary breast cancer samples from metastatic disease sites. Upper panels: ER-positive (*N* = 2 patients); Lower panels: ER-negative (*N* = 2 patients). Note that doxycycline dose-dependently (0, 25, 50 and 100 µM) inhibits mammosphere formation in four different primary patient samples derived from metastatic disease sites (either pleural effusions or ascites). Doxycycline works well in metastatic samples derived from both ER-positive or ER-negative patients. All experiments were performed in triplicate.
